# The impact of an infectious disease expert team on outpatient parenteral antimicrobial treatment in the Netherlands

**DOI:** 10.1007/s11096-018-0751-4

**Published:** 2018-11-26

**Authors:** Roos Wijnakker, Loes E. Visser, Emile F. Schippers, Leo G. Visser, Nathalie D. van Burgel, Cees van Nieuwkoop

**Affiliations:** 1 Department of Infectious Diseases, Leiden University Medical Center, Albinusdreef 2, 2333 ZA Leiden, The Netherlands; 2 Department of Hospital Pharmacy, Haga Teaching Hospital, The Hague, The Netherlands; 3 Department of Internal Medicine, Haga Teaching Hospital, The Hague, The Netherlands; 4 Department of Medical Microbiology, Haga Teaching Hospital, The Hague, The Netherlands

**Keywords:** Antimicrobial stewardship, Expert Consultation, Home treatment, Infectious diseases, OPAT, Outpatients, The Netherlands

## Abstract

*Background* There is increasing interest in outpatient parenteral antimicrobial treatment. *Objective* To evaluate the added value of consultation of an infectious diseases expert team (consisting of two internist-infectious diseases specialists and a microbiologist) for advice regarding type, administration route and duration of antibiotic treatment. *Setting* A retrospective case series was performed at the Haga Teaching Hospital, a 700-bed regional teaching hospital in The Hague, The Netherlands. *Methods* Complication rate and mortality was evaluated during 60 days of follow-up. Therapeutic rationality regarding outpatient parenteral antimicrobial treatment was determined by presenting randomly selected paper cases from the database to two independent infectious diseases specialists who were blinded to patient’s treatment and outcomes. The concordance between the two advices were analysed using Cohen’s kappa. For those with discordance, an infectious diseases expert team meeting was organized to reach consensus. The final recommendation was compared to the actual given antibiotic treatment. *Main outcome measure* Discrepancy between the infectious disease expert team recommendations upon type, administration route and duration of antibiotics and the real outpatient parenteral antimicrobial treatment practice. *Results* Out of 89 included cases, 50 were randomly selected for review by the infectious diseases specialists. The kappa statistic regarding antimicrobial policy was 0.581 (*P* < 0.001). In 78% (39/50 cases), they had complete agreement upon all aspects of antibiotic treatment. The remaining 11 cases were reviewed by the expert team. Comparing the consensus of 50 cases to actual practice, in 14(28%) cases there was a discrepancy suggesting potential room for improvement. Comparing the cases in whom an individual infectious diseases specialist was involved in real practice to those cases without, there was 18% versus 42% discrepancy with the recommendations of the expert team (OR 3.4; 95% CI: 0.9–12.5, *P* = 0.06). Complication rate was 19% including unplanned readmissions and side effects of antimicrobial agent or administration route. *Conclusion* Though outpatient parenteral antimicrobial treatment policies in the Netherlands appear to be safe, consultation of an ID expert team, rather than an individual ID specialist, has the potential to optimize antimicrobial treatment in patients considered suitable for outpatient parenteral antimicrobial treatment.

**Electronic supplementary material:**

The online version of this article (10.1007/s11096-018-0751-4) contains supplementary material, which is available to authorized users.

## Impacts on practice

- Outpatient parenteral antomicrobial treatment at home is generally safe with a high survival rate.
- Consultation of an infectious disease expert team can lead to a different diagnostic approach or treatment in the case of outpatient parenteral antimicrobial treatment (OPAT).

## Introduction

For many years inpatient care had been the standard for intravenous antimicrobial treatment and for many patients this is still needed to date. However, in 1974 Rucker et al reported their experience with outpatient parenteral antimicrobial treatment (OPAT) in children with cystic fibrosis [1, 2]. Since then, international interest has increased substantially and nowadays OPAT has become a more available modality for the treatment of many patients with a variety of infections [3–5].

OPAT is defined as the provision of parenteral antimicrobial therapy in at least two doses on different days without the need for hospitalization [5, 6]. It has been shown to be safe, effective and cost-saving due to decreasing length of hospitalization. Moreover, it enhances patient’s satisfaction, resulting in better quality of life [4, 7, 8]. The emergence of antimicrobial resistance limits the available oral antibiotic treatment options, especially for gram negative bacteria. As such, there is an increased need for OPAT while the individual patients become more complex with difficult to treat infections. Therefore, guidelines and checklists have been developed to allow a safe OPAT program, with the aim to prevent further development of antimicrobial resistance, both from an individual patient’s prospect as well as from a public health perspective [6, 9–11]. In this regard, consultation of a specialized multidisciplinary team, ideally being part of an antimicrobial stewardship team, has been proposed for every patient before the initiation of OPAT [10, 12–14].

In the Netherlands, OPAT is usually administered at home with daily skilled nursing visits reimbursed by the patient’s insurance. Although consultation of an infectious diseases (ID) specialist or pharmacist is advised by protocol, it is not mandatory. As such, consulting a multidisciplinary expert team for recommendations upon diagnostic approach, duration and type of administration route of OPAT, is not common practice.

## Aim of the study

The aim of the present study was to review the OPAT practice of a large regional teaching hospital, and to evaluate the impact of ID expert team consultation on the antimicrobial treatment plans.

## Methods

### Study population

A retrospective case series was performed at the Haga Teaching Hospital, a 700-bed regional teaching hospital in The Hague, The Netherlands. The selected patient population consisted of all adult patients who were discharged from the hospital with OPAT between January 1, 2010 and December 31, 2013. Patients were identified from a list of OPAT requests available at the pharmacy. By hospital protocol, the OPAT policy applied was that only patients who have met the following conditions are eligible to receive OPAT at home: (1) Patient has given informed consent; (2) Patient is able to visit the outpatient clinic of the attending specialist for routine control; (3) A peripherally inserted central catheter (PICC) has been placed; (4) The minimum OPAT duration is 7 days; (5) A once daily home visit by a specialized nurse is sufficient to administer the treatment; (6) IV antibiotics can be prepared by the specialized nurse at home. In addition, the general recommendation was to consult an ID specialist for advice upon type of antibiotic, possible oral alternatives, dose and duration of therapy. We excluded patients aged < 18 years, cystic fibrosis patients and patients with missing data essential for review. In case a patient had multiple OPAT requests during the study period, only the first OPAT request was included in the study.

### Data collection

Patient data were collected from the electronic patient records supplemented with details from the pharmacy and medical microbiology records. The extracted variables included demographics (age, sex), history of allergies, renal function (estimated glomerular filtration rate), infectious disease(s) diagnosis and treatment details (type of antibiotic, administration route, dosage and duration), microbiological test results and clinical outcome. The following outcome parameters were collected from the electronic patient records: (1) complications related to antibiotic treatment (allergic reactions and side effects) or administration route (replacement of PICC due to dislodgement and clinically diagnosed catheter related infections); (2) readmissions because of relapse during 60-days follow-up after discontinuation of OPAT, in which relapse is defined by a positive culture (e.g. blood, urine) in combination with symptoms and signs indicating relapse of the initial infection; (3) all-cause mortality during 60-days follow-up.

### Evaluation of the impact of a multidisciplinary infectious diseases expert team

Out of the selected patient’s database, 50 cases were randomly selected for independent review by the ID expert team in 2015. Details upon each case were anonymously described including age, sex, past medical history, history of allergies, kidney function, clinical infectious disease diagnosis and microbiological test results including susceptibility to antibiotics (see Supplement for a paper case example). Subsequently, these paper cases were reviewed by two ID specialists (EFS, Internist-ID specialist working at the Haga Teaching Hospital and the LUMC and LGV, Internist-ID specialist and professor of medicine at the LUMC) who were asked to give their personal specific recommendations upon choice of antibiotic, administration route (oral or intravenous), dosage and duration of treatment. These reviewers were not involved in the patient’s management and they were blinded to each other and to all other patient’s data including details upon the actual given antimicrobial treatment. Comparing the recommendations of these reviewers, it was assumed that the optimal antimicrobial treatment of each case should have been their recommendation when they fully agreed upon all aspects. In case of inter-observer disagreement, a consensus meeting with two ID specialists (EFS and CvN) and one medical microbiologist (NDvB) was organized, to achieve agreement upon the optimal treatment. This team was chosen as by the time of the consensus meeting these specialists were all members of the antimicrobial stewardship team of the Haga Teaching Hospital which has started in 2014. The final consensus recommendations of this expert team were considered a simulation of advice from a multidisciplinary ID expert team. Finally, the recommendations based on the reviewed paper cases were compared to the actual treatment of each patient, to evaluate the potential room for improvement.

### Statistical analysis

Descriptive analysis included means with standard deviation (SD), percentages or medians and ranges, as appropriate. Chi square tests were used to evaluate differences between binary outcomes.

The inter-observer agreement between the two reviewers was analyzed using Cohen’s kappa test. A value of 0.41–0.60 corresponds to fair or moderate agreement, 0.61–0.80 corresponds to good agreement and 0.80–1.0 corresponds to very good or almost perfect agreement [15, 16].

All statistical tests were performed using IBM^→^ SPSS^→^ Statistics, version 24.

## Ethics approval

As this was a retrospective observational quality study, the Institutional Scientific Review Board and the Medical Ethics Committee of the Haga Teaching Hospital approved the study and waived the need for patient’s informed consent (Protocol number 15-020).

## Results

**Fig. 1:**
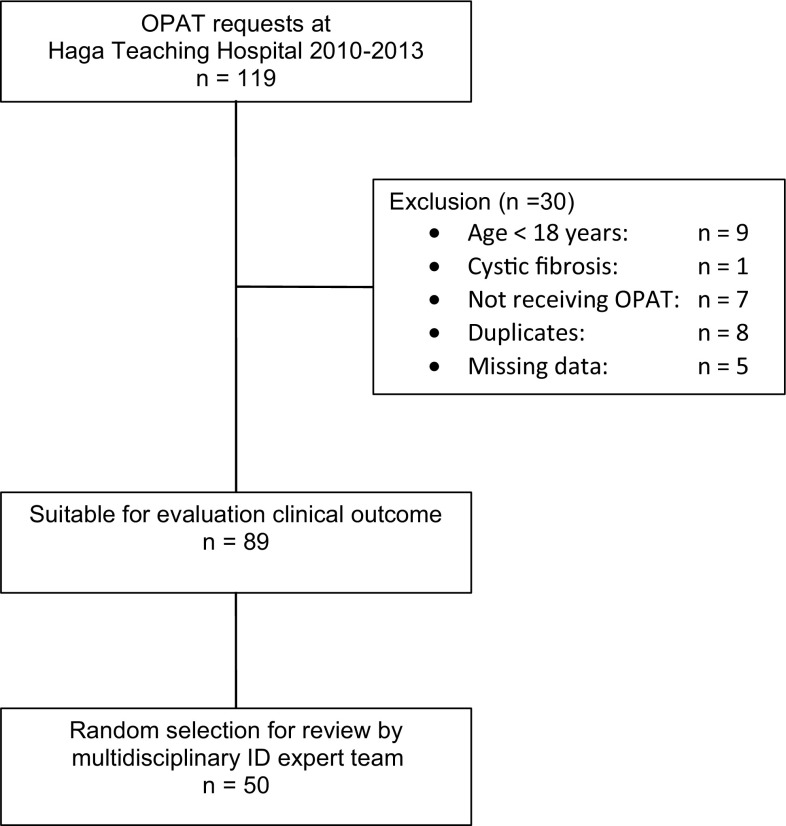
Flow chart of patient selection

From January 2010 to December 2013, there were 119 requests for OPAT in the home setting via the Haga Teaching Hospital. Of these 119, 30 were excluded from analysis for various reasons (Fig. 1).

The baseline patient characteristics of the study population are listed in Table 1. Most cases were treated at the internal medicine department (47.2%); a limited number of OPAT requests originated from the cardiology or neurology department. A formal ID consult was obtained in 39.3% before discharge. The median duration of OPAT was 28 days.

**Table 1:** Baseline patient characteristics

Variable	Total population (n = 89)
Male sex (n, %)	57 (64)
Age (median + range)	66 (20–87)
Department (*n*, %)	
Internal Medicine	42 (47.2)
Urology	14 (15.7)
Orthopedics	12 (13.5)
Clinical diagnoses (*n*, %)	
Bone and Joint Infections	35 (38.1)
Septic Arthritis	13 (14.6)
Spondylodiscitis	10 (11.2)
Prothetic joint infections	8 (8.9)
Osteomyelitis	4 (4.5)
Urinary tract infections	25 (28.1)
Pyelonefritis	9 (10.1)
Prostatitis	5 (5.6)
Urosepsis	11 (12.3)
Community acquired *Staphylococcus aureus* bacteremia	10 (11.2)
Abscess	5 (5.6)
Endocarditis	4 (3.3)
Other (bacteremia, meningitis, neuroborreliosis, neurosyphilis)	10 (11.2)
Antimicrobial diagnoses (*n*, %)	
None	10 (11.2)
*Staphylococcus aureus*	24 (26.9)
*Escherichia coli*	21 (23.6)
*Staphylococcus epidermidis*	3 (3,4)
*Klebsiella pneumonia*	3 (3.4)
*Haem. Streptococcus A/B/C*	5 (5.6)
Other (*S. Milleri*, *S. pneumonia*, *corynebacterium*, *E. faecalis)*	16 (17.9)
* Polymicrobial*	8 (8.9)
Multiresistant pathogen (%)	19 (21.3)
Formal ID consult (*n*, %)	35 (39.3)
Type of antimicrobial used (%)	
Flucloxacillin	26 (29.2)
Carbapenems	15 (16.9)
Ceftriaxon	15 (16.9)
Benzylpenicillin	12 (13.5)
Cefuroxim	10 (11.2)
Vancomycin	7 (7.9)
Ceftazidim	2 (2.2)
Cefalozin and others	2 (2.2)
Duration OPAT treatment (median +IQR)	28 days (14–47)

Primary conditions treated with OPAT were bone and joint infections, urinary tract infections and community acquired *Staphylococcus aureus* bacteremia (Table 1). Endocarditis was underrepresented in this study, with 3.3% of total requests. The most frequent involved pathogens were *S. aureus* (26.9%) and *Escherichia coli* (23.6%). There were no cases with methicillin resistant *S. aureus* (MRSA). Flucloxacillin sodium was used in 29.2% of OPAT patients, with bone and joint infections and community acquired *S. aureus* bacteremia as the main indications. Carbapenems and Ceftriaxone were the second and third most prescribed antibiotics for the treatment of urinary tract infections.

Of the 89 studied patients, 17 (19%) patients presented with complications during follow up; 9% were antibiotic related (3 patients with allergy, 4 with adverse effects), 6.7% related to administration route (3 patients needed replacement of PICC/peripheral administration route, 2 patients with infected PICC and 1 patient with pneumothorax), and 3.3% were readmitted because of relapse within 60 days after the end of treatment. The 60-day survival was 96.6% after discharge with OPAT. Two (2.2%) patients died during OPAT; one patient died because of deterioration related to the infection and another patient passed away suddenly at home. This cause of death was considered unrelated to OPAT or the underlying infection as judged by the family doctor.

**Fig. 2:**
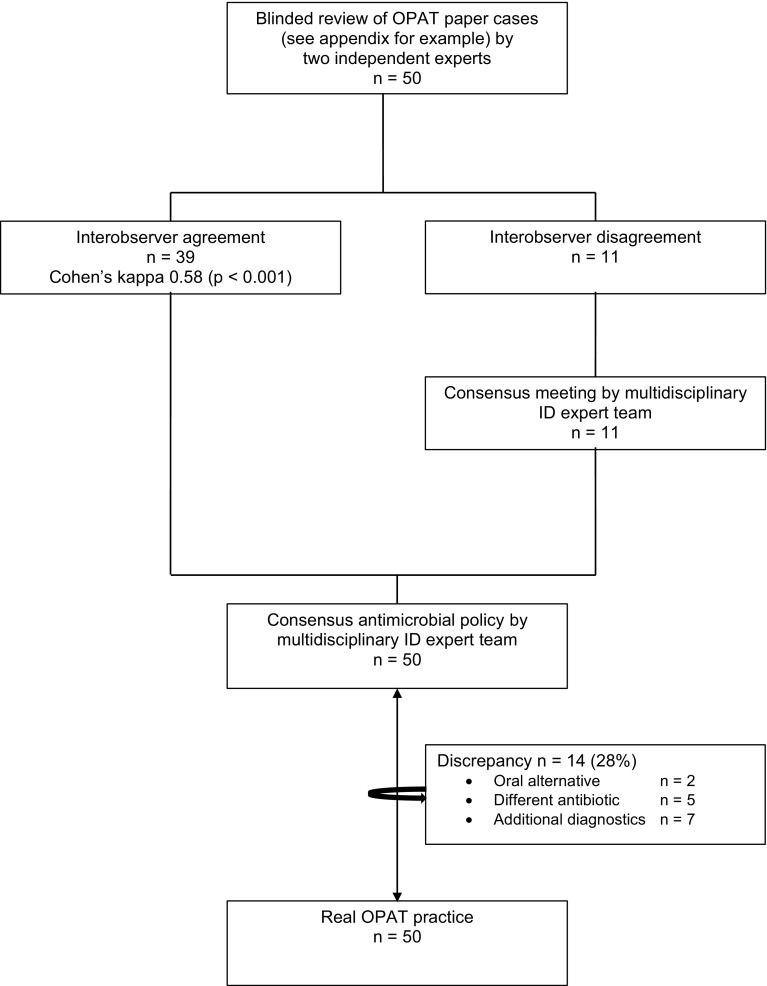
Potential room for improvement of OPAT by multidisciplinary ID expert team

To evaluate the therapeutic appropriateness of OPAT, 50 out of the 89 cases were randomly selected and presented to two independent ID specialists (Fig. 1). In 39 (78%) of the presented cases, they agreed upon all aspects of antibiotic treatment. The interobserver agreement regarding spectrum and route of antibiotic treatment was 0.581 (moderate agreement, *P* < 0.001). For the dose and duration of antibiotic treatment the Cohen’s kappa was respectively 0.404 (fair agreement, *P* < 0.001) and 0.524 (moderate agreement, *P* < 0.000). The remaining 11 cases with discrepant recommendations were then discussed in a multidisciplinary ID expert team to achieve consensus. Comparing the final recommendations of the expert team upon all 50 cases to the actual given treatment, there was a discrepancy, and thus room for improvement, in 14 cases (28%) (Fig. 2). In 2 cases an oral alternative antibiotic was advised, in 7 cases further diagnostic tests were deemed necessary to determine a rational therapeutic advice and in the remaining 5 cases the expert team recommended a different intravenous antibiotic drug. In 3 of these cases a narrow spectrum intravenous antibiotic was advised and in 2 cases a different generation of cephalosporin. In real practice, in 22 of the 50 randomly selected cases an ID specialist was consulted to give advice about the optimal antimicrobial treatment. Comparing the cases in whom an ID specialist was involved to those cases without, there was 18% versus 42% discrepancy with the recommendations of the infectious diseases expert team (OR 3.4; 95% CI: 0.9–12.5, *P* = 0.06, not statistically significant).

## Discussion

In this study, we evaluated the clinical outcome of patients with OPAT at home and the potential impact of consultation of a multidisciplinary ID expert team on treatment policy. We found that in 28% of cases, consultation of an expert team might have led to a different diagnostic approach or treatment. In addition, we found that OPAT at home is generally safe with a high survival rate. Furthermore, we showed that ID experts have moderate interobserver agreement upon their antimicrobial treatment advice.

The latter is comparable to a recent study about expert’s agreement upon the optimal antibiotic therapy showing a Cohen’s kappa ranging from 0.32 to 0.72 [17]. In our approach, all cases with disagreement between experts were reviewed by a multidisciplinary expert team. In our opinion, such a multidisciplinary expert team should ideally consist of a medical microbiologist, two ID specialists (i.e. medical doctors) and a pharmacist reflecting an antimicrobial stewardship team [17, 18].

Methicillin-sensitive *S. aureus* was the most frequent involved pathogen in this study and consequently flucloxacillin sodium was the most frequent used antibiotic. As there were no cases with MRSA in our study, this is different to other studies with vancomycin being used more often [2, 5, 19, 20]. Endocarditis appeared to be underrepresented in this study with only 3.3% of the cases reflecting the policy of our department of cardiology at that time with strong preference to treat patients with infective endocarditis in the hospital. Seaton et al showed that after bone and joint infections, endocarditis was the third most common indication treated with OPAT [5]. The average duration of OPAT in this study was generally comparable to practices in other countries [3].

A formal ID consultation was performed in 39% of the cases. Previously, an ID consult was shown to result in a more safe and effective OPAT, with less incorrect use of intravenous antibiotic therapy and thus saving costs [8, 11, 12, 19]. Furthermore, an ID consult might prevent OPAT in 10% of all requests without any impact on the patient’s clinical outcome [13]. Moreover, comparing the cases without an individual ID consult in real practice to the cases with an ID consult, the cases without an ID consult had more potential room for improvement (42% vs 18%). This is comparable with a recent study showing about 20% discrepancies between individual ID experts and the reference standard [17]. As the advice of an individual ID specialist is suboptimal in about 20% of the cases, this should be an argument for standard consultation of a multidisciplinary ID expert team when OPAT is considered.

Earlier studies have shown a complication rate up to 25%, whereas in this study it was 19% [2, 12, 21]. Perhaps, due its retrospective nature, complications were underreported in this study. In addition, only unplanned readmissions because of proven relapse were considered complications, whereas in previous studies all readmissions were included as such [20, 22]. Mortality during OPAT was similar to previous studies, being around 2%.

Our study has some limitations due to its retrospective design. We only included patients who actually received OPAT at home. Though it was not the scope of this study, it might well be that other patients who were treated with intravenous antibiotics in the hospital, were also good candidates for OPAT (e.g. patients with endocarditis). Whether consultation of an ID expert team in such patients would also have potential room for improvement, is of interest. Furthermore, though the complication rate was comparable with previous studies, we are not able to evaluate whether OPAT actually has improved the clinical outcome of the patients as there was no control group and patient’s satisfaction was not measured. Nevertheless, it should be noted that the evaluation of the impact of a multidisciplinary ID expert team on antimicrobial treatment policy was an independent blinded part of this study. However, whether consultation of such an expert team indeed would have led to better patient outcomes could not be determined by this study. Previous studies have shown the added value of a bedside ID consult. In this study, only paper cases were reviewed. This study includes the OPAT practice of 2010–2013 and the ID expert team reviewed the cases in 2015. Although OPAT have changed over the years, we consider the data presented in this article are still representative for the patients treated to date. Ideally, a randomized trial comparing the use of an ID expert team versus no use should be performed to evaluate its impact on patient’s outcome with OPAT.

## Conclusion

This study indicates that although OPAT policies in the Netherlands appear to be safe, consultation of an ID expert team, rather than an individual ID specialist, has the potential to optimize antimicrobial treatment in patients considered suitable for OPAT. Implementation of a standard ID expert team consultation could lead to prescriptions of more narrow antimicrobial treatment, shorter duration of treatment and better diagnostic approach. This could contribute to the goals of antibiotic stewardship.

## Electronic supplementary material

Below is the link to the electronic supplementary material.

Supplementary material 1 (DOCX 18 kb)
