# Supplementary material for: The impact of an infectious disease expert team on outpatient parenteral antimicrobial treatment in the Netherlands
Source: Int J Clin Pharm. 2018 Nov 26;41(1):49–55. doi: 10.1007/s11096-018-0751-4 (PMC6394504; doi:10.1007/s11096-018-0751-4)
Supplement: Supplementary file 1 — Supplementary material 1 (DOCX 18 kb) [file 11096_2018_751_MOESM1_ESM.docx]

**Supporting information: Example paper case presented to the antimicrobial stewardship team**

**♀ X**

*Date of birth:* 25.09.1974

*Date of admission: 08.05.2013*

Background:

Recently treated urinary tract infection with cefuroxim and ciprofloxacin. Readmission because of recurrent fever and right-sided costovertebral tenderness.

History:

- Autosomal dominant polycystic kidney disease
- Hypertension
- Obesity
- Hypercholesterolaemia

Medication used at presentation:

- Hydrochloorthiazide/perindopril, propranolol, simvastatin, amlodipine

Physical examination:

Pulse 83/min, Blood pressure 117/64 mm Hg, Temp 37.3 ^o^Ce, Respiratory rate 12/min

Infection disease related essentials:

1. **Infection diagnosis**: Renal cyst infection
2. **Antimicrobial diagnosis**: Urine culture: *Escherichia coli* (ESBL negative) and *haemolytic streptococ* group B
3. **Antibiogram** **of *E.coli***:

Amoxicilline R, Amoxicillin-Clavulanic acid S, Ceftriaxon S, Cefuroxim S, Ciprofloxacin R, Carbapenems S

Trimethoprim-Sulfamethoxazole R, Fosfomycin S, Nitrofurantoin S, Tobramycine S

1. **Allergy**: none
2. **eGFR (MDRD)**: 59ml/min/1.73m^2^

Questions:

1. What is the optimal antimicrobial treatment in this case?
2. What is the best route, dose and duration that should be used?
3. Would it be possible to use OPAT in this case?
